# Christian Science and the Recent Inquest

**Published:** 1910-02-12

**Authors:** Algernon Hervey-Bathurst

**Affiliations:** Assistant. 23 and 24 Clun House, Surrey Street, Strand, W.C.


*To the Editor of* The Hospital.

### CHRISTIAN SCIENCE AND THE RECENT INQUEST.

*To the Editor of* The Hospital.

Sir,—Will you allow me to refer to a report which appeared in your columns of the inquest on Captain Donne? In the evidence it was stated that Captain Donne had consulted no fewer than twenty-two doctors before finally turning to Christian Science. All of these doctors had pronounced his case to be incurable, and, indeed, the last one consulted, and that was eight years ago, gave the patient only three months to live. In view of the above facts there is not the slightest ground for the statement that "if Captain Donne had been under medical treatment during the whole of his illness his life might have been prolonged for another year." In the same manner, the expression of regret from the gentlemen of the jury, "that no medical man was called in," cannot be justified. A careful study of the facts will show that there is but one logical conclusion to be drawn, namely, that it was entirely to Christian Science that Captain Donne owed the last eight years of his life.

In conclusion, let me point out that the patient was not treated continuously during seven years by Mrs. Braithwaite, but only at intervals during that period.—Yours truly,

ALGERNON HERVEY-BATHURST, Assistant.
23 and 24 Clun House, Surrey Street, Strand, W.C.
February 1, 1910.

